# Case Report: Post-transplant lymphoproliferative disorder as a serious complication of vascularized composite allotransplantation

**DOI:** 10.3389/frtra.2024.1339898

**Published:** 2024-03-14

**Authors:** Alessandra Zaccardelli, Fabienne M. Lucas, Ann S. LaCasce, Anil K. Chandraker, Jamil R. Azzi, Simon G. Talbot

**Affiliations:** ^1^ Department of Medical Education, Tufts University School of Medicine, Boston, MA, United States; ^2^ Department of Pathology, Brigham and Women’s Hospital, Boston, MA, United States; ^3^ Department of Medical Oncology, Dana-Farber Cancer Institute, Boston, MA, United States; ^4^ Department of Medicine, Brigham and Women’s Hospital, Boston, MA, United States; ^5^ Division of Plastic and Reconstructive Surgery, Brigham and Women’s Hospital, Boston, MA, United States

**Keywords:** vascularized composite allotransplantation, post-transplant lymphoproliferative disorder, rejection, immunosuppression, histopathology diffuse large B-cell lymphoma

## Abstract

Vascularized composite allotransplantation (VCA) is an emerging field in transplant surgery. Despite overall positive outcomes, VCA confers risk for multiple complications related to the procedure and subsequent immunosuppression. Post-transplant lymphoproliferative disorder (PTLD) is a heterogeneous group of lymphoproliferative disorders occurring after solid organ and hematopoietic stem cell transplant. A patient with PTLD after bilateral upper extremity transplantation is presented as well as a review of all known cases of PTLD after VCA, with a focus on the unique epidemiology, presentation, and treatment in this population.

## 1 Introduction

Vascularized composite allotransplantation (VCA) is the transplantation of multiple tissue types to restore form and function in patients with debilitating injuries when traditional treatments prove inadequate. Since the first successful hand transplant in 1998 (1), over 115 patients have undergone facial (43%) and upper extremity VCA (57%) (2). Despite overall positive outcomes for transplant survival (3), limb function (4), and psychosocial acceptance (5), VCA confers significant risk linked to the long-term immunosuppression required to prevent allograft rejection. Notably, we have recently observed post-transplant lymphoproliferative disorder (PTLD) in VCA patients. PTLD is a heterogeneous group of lymphoproliferative disorders observed in recipients of solid organ transplants (SOTs), hematopoietic stem cell transplants (HSCTs), and now, VCA (6–8). And while this is not unexpected, as the prevalence of VCA patients increases, practitioners need to be cognizant of the unique considerations relevant to diagnosis and treatment. In this report, we present a novel case of very late onset Epstein–Barr virus (EBV)–negative PTLD after bilateral upper extremity transplantation, review all known cases in VCA recipients, and discuss etiology, pathophysiology, and our treatment approach with an emphasis on how PTLD differs in VCA versus conventional transplants.

## 2 Patient information

**Figure 1:**
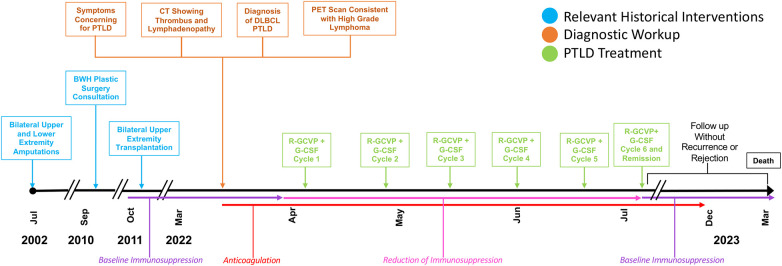
Timeline with relevant timepoints from the episode of care. PET, positron emission tomography.

Our patient is a 65-year-old man who underwent bilateral upper (below-elbow) and lower extremity (below-knee) amputations in July 2002 secondary to urosepsis complicated by acute respiratory distress syndrome resulting in gangrene of the hands and feet (Figure 1). The patient was referred to Brigham and Women's Hospital Plastic Surgery Service for consideration of upper extremity allotransplantation due to dissatisfaction with his arm prostheses. He underwent extensive screening before being listed for allotransplantation, including preoperative testing with transplant medicine, renal, infectious disease, critical care, psychiatry, social work, and rehabilitation services, as well as pre-transplantation laboratory testing, electromyography/nerve conduction studies, and imaging of both upper extremities with plain films and angiograms. Serum enzyme immunoassay was negative for cytomegalovirus (CMV) IgG and IgM, positive for EBV-IgG, and negative for EBV-IgM. In early October 2011, a suitably matched CMV-negative deceased donor was identified. T- and B-cell crossmatching was negative and human leukocyte antigen (HLA)-typing showed mismatching at the A, B, and DR loci (recipient antigens: A3 A11 B7 B13 B7 DR7 DR15, donor antigens: A2 B8 BW60 DR4 DR13).

In October 2011, 2 days after donor identification, the patient underwent bilateral upper extremity transplantation. The operative course was relatively unremarkable. Cold ischemia time was under 4 h. Due to right hand ischemia in the 6 h after surgery, the patient returned to the operating room for thrombectomy and re-anastomosis of the radial and ulnar arteries. Low-dose intravenous (IV) heparin was used perioperatively. The patient received a total of seven units of packed red blood cells (RBCs) intraoperatively and two additional units postoperatively. Surgical site prophylaxis consisted of a 4-day course of linezolid (600 mg IV daily) and cefazolin (2 g IV every 8 h).

Induction immunosuppression used four doses of IV anti-thymocyte globulin (1.5 mg/kg). Maintenance immunosuppression was initiated with tacrolimus (trough 10–15 ng/ml), mycophenolate (1,000 mg twice daily), and prednisone (7.5 mg daily). CMV and pneumocystis pneumonia prophylaxis used valacyclovir (500 mg twice daily) and trimethoprim–sulfamethoxazole (one tablet daily), respectively. Tacrolimus and mycophenolate were gradually weaned to a goal tacrolimus trough of 5 ng/ml and 360 mg twice daily, respectively.

Two episodes of Banff grade II–III rejection occurred during the winter months of 2013 and 2014 (post-transplant months 26 and 37, respectively). The first was treated with topical tacrolimus, plus increased oral mycophenolate and tacrolimus. The second resolved with topical tacrolimus, clobetasol, and increased oral tacrolimus. Lymphodepletion was not required due to these initial treatments being effective. We believe that extreme cold, low humidity, and microtrauma may have been instigating rejection. Prednisone (5 mg daily) was initiated each winter for the prevention of further rejection episodes.

## 3 Clinical findings

During the year-11 annual post-transplant clinic visit, in March 2022, the patient reported intermittent night sweats, shortness of breath, and left upper extremity swelling. A physical examination demonstrated asymmetrical left upper extremity swelling and bilateral cervical lymphadenopathy.

## 4 Diagnostic assessment

**Figure 2:**
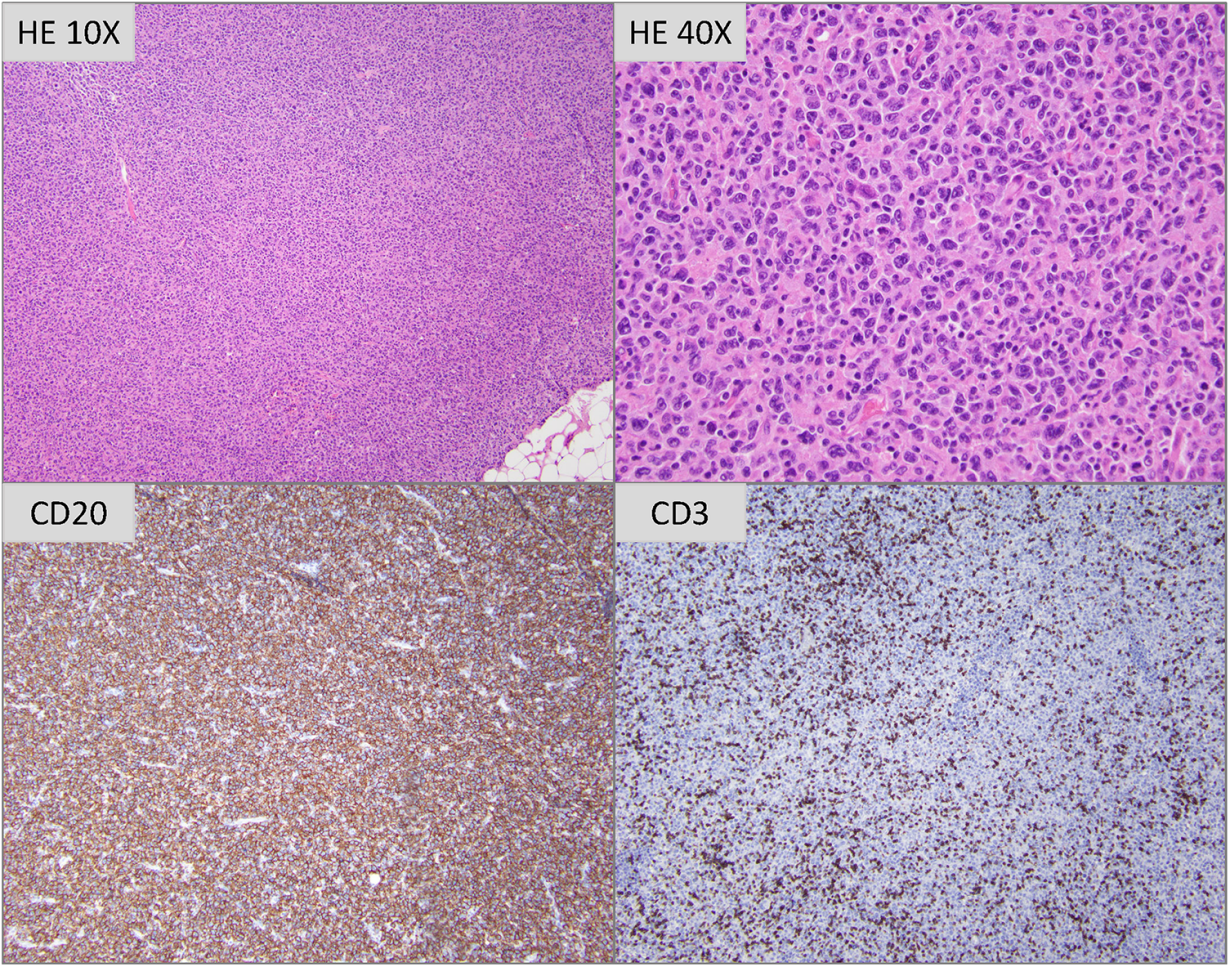
Hematoxylin and eosin stained tissue sections (photomicrographs taken with 10× and 40× objectives are depicted) show profiles of lymph node with architecture completely effaced by a polymorphous infiltrate of atypical lymphoid cells of variable size and morphology. Most are intermediate to large sized cells with irregular nuclei, vesicular to open chromatin, distinct nucleoli and moderate amounts of eosinophilic cytoplasm. Interspersed scattered large sized multinucleated cells with large amounts of cytoplasm are seen. Immunoperoxidase studies reveal that the atypical cells are CD20-positive B cells, with frequent small sized CD3-positive T lymphocytes admixed.

Chest and neck computed tomography (CT) showed an anterior mediastinal mass with cervical lymphadenopathy causing subclavian vein compression, as well as a small thrombus at the junction of the left internal jugular and subclavian veins. The patient was admitted for anticoagulation using Eliquis and an excisional axillary lymph node biopsy demonstrating effacement by atypical lymphoid cells of variable size and morphology (Figure 2). Immunoperoxidase and *in situ* hybridization studies revealed that the atypical cells were B cells positive for cluster of differentiation (CD) 20, PAX5, B-cell lymphoma (BCL) 2, BCL6 (>80% of atypical cells) and MYC (>50% of atypical cells), and negative for CD5, CD10, multiple myeloma oncogene-1 (10%–20% positive atypical cells, interpreted as negative), CD30, CD15, and Epstein–Barr virus–encoded RNA, with a Ki67 proliferative index of 60%–70%. Given transplantation, a diagnosis of “monomorphic post-transplant lymphoproliferative disorder (M-PTLD), morphologic features of Diffuse Large B-cell Lymphoma (DLBCL), germinal center B-cell subtype” was rendered per the 2017 World Health Organization (WHO) classification revised 4th edition (HAEM4R) (7). This diagnosis is unchanged using the 2022 International Consensus Classification system (9); the corresponding diagnosis using the WHO5 classification (6) would be “Diffuse Large B-cell Lymphoma, germinal center B-cell subtype, EBV-negative, post-transplant”, applying a three-part nomenclature capturing histological diagnosis, viral association, and immune deficiency/dysregulation setting. A positron emission tomography scan demonstrated intense fluorodeoxyglucose-avid lymphadenopathy predominantly above, but also below, the diaphragm in the gastrohepatic lymph nodes, consistent with aggressive lymphoma and a small pericardial effusion.

## 5 Therapeutic intervention

Tacrolimus was discontinued, and mycophenolate was decreased to 180 mg once daily then discontinued. Given the patient was not a candidate for anthracycline-containing therapy due to baseline cardiomyopathy with reduced ejection fraction (34%), he was treated with six cycles of rituximab, gemcitabine, cyclophosphamide, vincristine, and prednisone (R-GCVP) with granulocyte colony-stimulating factor (G-CSF). In July 2022, the patient demonstrated evidence of complete remission. Immunosuppression was restarted, substituting sirolimus for tacrolimus to maximize its anti-tumor properties.

## 6 Follow-up and outcomes

Anticoagulation was discontinued after 5 months when vascular ultrasound showed no further thrombus present. No signs of rejection or graft compromise were detected during the treatment phase or on post-treatment surveillance biopsy. The patient died 1 year after the PTLD diagnosis from unrelated cardiovascular disease.

## 7 Discussion

VCA is a surgical option to restore form and function to a subset of patients experiencing otherwise unreconstructible tissue loss. Despite generally favorable outcomes, VCA confers a substantial risk of complications related to surgery and immunosuppression (2). As the rates of VCA increase, we expect complications to be more frequent. Thus, it is prudent to draw awareness to adverse outcomes, especially those that are considered rare in VCA and are therefore often overlooked, prompting this case report.

PTLD is well studied among recipients of SOTs and HSCTs, with an estimated incidence of 2%–20% among transplant patients (10). By contrast, to date only three cases of PTLD after VCA have been documented (1, 11, 12) (Table 1). The first was reported by the International Registry on Hand and Composite Tissue Transplantation in an analysis of all cases of hand transplant with follow-up between 2002 and 2010. Of the 33 recipients, one patient who underwent unilateral hand transplant in 2006 subsequently developed PTLD (1). No other details of the case were included in the report.

**Table 1:** Reports of PTLD in VCA recipients.

Date of VCA (month, year)	Type of VCA	Age at time of VCA (years)	Immunosuppression	Time of PTLD Diagnosis (postoperative month)	Type of PTLD	Treatment	Outcome	Reference (author, year)
2006	Unilateral hand	–	–	–	–	–	–	Petruzzo et al. (1)
November 2009	Face	27	Induction: antithymocyte globulin Maintenance: tacrolimus, mycophenolate mofetil, and steroids	5 Recurrence at month 15	EBV-associated DLBCL	Initial treatment: rituximab, reduction of tacrolimus, discontinuation of mycophenolate Recurrence treatment: eight cycles of R-CHOP; cessation of tacrolimus and mycophenolate	August 2011 (month 21): complete remission April 2017 (month 89): development of EBV-associated post-transplant smooth muscle tumors leading to liver transplant in April 2017 October 2017 (month 95): pneumonia and sepsis leading to death	Petruzzo et al. (12)
July 2011	Bilateral lower extremity	22	Induction: alemtuzumab Maintenance: tacrolimus, mycophenolate mofetil, and prednisone	15	EBV-associated PCNS	Discontinuation of immunosuppression Explantation	Complete remission	Cavadas et al. (11)

CMV, cytomegalovirus; DLBCL, diffuse large B-cell lymphoma; EBV, Epstein–Barr virus; PCNS, primary central nervous system; PTLD, post-transplant lymphoproliferative disorder; R-CHOP, rituximab, cyclophosphamide, doxorubicin, vincristine, and prednisone; VCA, vascularized composite allotransplantation.

The second case was observed in a 27-year-old who received a facial allograft, including an edentulous mandible, upper and lower lips, cheeks, and chin, as well as a donor sentinel vascularized flap to the abdomen in November 2009 (12). Induction immunosuppression was treated with anti-thymocyte globulin; tacrolimus, mycophenolate mofetil, and steroids were used for maintenance. The follow-up was notable for numerous oral herpes simplex virus type 1 infections with the development of acyclovir resistance, as well as primary asymptomatic EBV infection in April 2010. The patient then developed EBV-associated DLBCL treated with rituximab as well as a decrease in the tacrolimus dose and the discontinuation of mycophenolate with the induction of remission. After reintroducing mycophenolate, the patient relapsed. Treatment included eight cycles of rituximab, cyclophosphamide, doxorubicin, vincristine, and prednisone with cessation of tacrolimus and mycophenolate. Complete remission was achieved in August 2011 and tacrolimus was reinitiated. Subsequent hepatic EBV-associated smooth muscle tumors required a reduction of immunosuppression. These periodic pauses in immunosuppression likely contributed to the ensuing episodes of acute and chronic rejection (Banff grade III at 18 and 24 months with features outside the Banff classification). Despite persistent rejection and graft dysfunction, the patient opted to keep the graft without PTLD recurrence. Of note, the progression of EBV-associated post-transplant smooth muscle tumors led to a liver transplant in April 2017. The patient died in October 2017 of pneumonia, sepsis, and consequent multiorgan failure (13).

The third case was one of primary central nervous system (PCNS) PTLD in a 22-year-old bilateral transfemoral lower extremity transplant recipient (11). In July 2011, this patient underwent lower limb transplantation; serostatus was CMV-negative and EBV-negative for both donor and recipient. Induction used alemtuzumab; tacrolimus, mycophenolate mofetil, and prednisone were used for maintenance. When tacrolimus was exchanged for sirolimus to reduce the risk of malignancy, the patient developed CMV as well as acute skin rejection (Banff grade I), requiring intravenous valganciclovir and intravenous methylprednisolone. Acute rejection persisted, necessitating intravenous immunoglobulin and a transition from sirolimus to tacrolimus facilitating rapid recovery. Acyclovir was discontinued nearly 1 year after the initiation of treatment when CMV immunity was verified. At month 15, the patient was noted to have continued improvement of limb function but then developed strabismus, diplopia, and hypertropia of the left eye; magnetic resonance imaging revealed a brainstem lesion. Stereotactic biopsies demonstrated EBV-positive PTLD with large pleomorphic lymphoid cells. The patient elected to discontinue immunosuppression and underwent explantation of his transplanted limbs to maximize treatment consisting of methotrexate and stereotactic radiotherapy. The patient achieved complete remission, which was maintained at follow-up 23 months after diagnosis. To our knowledge, this patient is alive at the time of publication.

PTLD arises from immune suppression and includes EBV-positive and -negative disease. Early lesions are typically polyclonal, EBV-associated, and seen in pediatric patients. EBV mismatch is a major driver of PTLD, with the highest risk in EBV-negative recipients of EBV-positive donor grafts. The PTLD risk correlates with the intensity and duration of immune suppression and specific agents, including tacrolimus (14), anti-CD3 (15), and cyclosporine (16, 17). In addition, PTLD rates differ depending on the organ transplant, partly due to the extent of graft lymphoid tissue (12). Other proposed risk factors include donor or recipient HLA-A26 and HLA-B38 haplotypes (18) and certain cytokine polymorphisms (19–21). EBV-negative PTLD occurred in our patient who was receiving long-term tacrolimus, mycophenolate, and prednisone. This underscores that VCA patients, who may receive multiple episodic treatments for acute rejection, may be vulnerable even in the absence of other risk factors. Our patient also demonstrates the potential role of acute and chronic rejection in PTLD development. In particular, the early episodes of acute rejection required multiple rounds of elevated immunosuppression potentially setting the stage for dysregulated cellular proliferation.

In general, PTLD follows a bimodal distribution of high incidence within the first year after transplant and a second spike 5 years after transplant (“late onset”) (13), with some variability depending on transplant type and PTLD subtype. In particular, a more rapid onset has been observed among EBV-positive versus EBV-negative SOT recipients (10 vs. 50 months) and HSCT recipients. The only other cases of PTLD in VCA patients have followed this time course: the cases of EBV-positive B-cell lymphoma (12) and PCNS PTLD (11) presented 6 and 10 months postoperatively, respectively. Rarely, PTLD can occur more than 10 years after transplant (“very late onset”), a phenomenon that has been associated with older age and treatment with tacrolimus alone or in combination with mycophenolate or azathioprine in SOT patients (22). Our patient demonstrates a rare case of very late onset PTLD, presenting just over 10 years after transplant. In contrast to prior cases of PTLD in VCA, which may have been hastened by EBV infection or demographics such as younger age, our elderly patient had EBV-negative PTLD, highlighting the need for ongoing vigilance in this patient population. This may also have implications for the management of VCA patients, whose risk of very late PTLD may increase based on the specific immunosuppressant agents, which, unfortunately, we lack the ability to tailor.

Given the risks intrinsically associated with long-term high-dose immunosuppressants, surveillance for PTLD has been recommended, particularly in high-risk individuals with EBV mismatch. Measurement for EBV viral load may detect early PTLD, and EBV viremia indicates imaging to assess active lymphoma. Pre-emptive rituximab plus immunosuppression reduction has been trialed among patients with EBV reactivation or infection. However, the optimal testing and interpretation of EBV viral load remains unclear. Among high-risk SOT patients, serial monitoring of the EBV viral load has demonstrated high sensitivity but low specificity for the detection of PTLD (23). This method of screening may be of benefit for many VCA patients but is unfortunately ineffective for the detection of EBV-negative disease. As VCA patients may be vulnerable to PTLD in the absence of classical risk factors, such as EBV mismatch, even those considered low-risk should be counseled to monitor for signs of PTLD development.

The clinical presentation of PTLD is non-specific and highly variable. The presenting symptoms may include B-symptoms resembling mononucleosis (10), as well as night sweats, weight loss, and lymphadenopathy, as in our patient (24). Extranodal involvement is common, often involving the transplanted organ in SOT. In VCA recipients, lymphadenopathy can be an issue. First, it may be challenging to diagnose as lymph nodes are part of the allograft (as in face transplants) and are prone to benign lymphadenopathy (such as with recurrent minor infections of the exterior allograft). Second, lymphadenopathy may impair venous return from the allograft, presenting as venous thrombus, venous thromboembolism, and edema or venous congestion, as demonstrated by our patient's internal jugular/subclavian thrombus.

The mainstay of PTLD treatment is the reduction of immunosuppression with the primary goal of inducing remission with the preservation of transplanted tissue (25). This is typically achieved via the discontinuation of antimetabolic agents, such as mycophenolate or azathioprine, and a 50% reduction of calcineurin inhibitors. For monomorphic DLBCL, a sequential approach is often pursued with single agent rituximab followed by response-adapted therapy with additional rituximab in patients achieving complete response and chemotherapy (typically cyclophosphamide, doxorubicin, vincristine, and prednisone) (26). Though effective, the reduction of immunosuppression confers a substantial risk of acute or chronic rejection in SOT and HSCT patients, with reported rates of acute rejection as high as 40% (17). Rejection while on rituximab and chemotherapy is rare but can occur with the waning of the immunosuppressive effect. Unsurprisingly, this risk extends to VCA patients as well; in the facial graft recipient above, this ultimately led to advancement from acute to chronic rejection characterized by progressive sclerosis, hyperpigmentation, and graft dysfunction (12). Indeed, the reduction of immunosuppression and ensuing graft deterioration poses a significant threat to VCA patients who require substantial immunosuppression. Thus, monitoring for rejection is essential throughout PTLD treatment, as in our patient who was followed closely for clinical signs of change, including rashes, changes in the sensitive eponychial folds, and ischemic or necrotic ulcerations. This method is invasive and tends to detect rejection later in the rejection process. Recently proposed methods to survey for rejection in VCA include ultrasound biomicroscopy and functional magnetic resonance imaging. These modalities require further validation but show promise to detect early rejection, which may be of particular benefit during the reduction of immunosuppression (27). An alternative budding strategy is the measurement of serum biomarkers, such as matrix metalloproteinase-3 or donor-derived microparticles, which have been shown to rise with rejection in preliminary studies among VCA recipients (28). However, the determination of accurate biomarkers has been challenging in this population due to the small number of VCA patients. Once PTLD is controlled, the rapid reintroduction of immunosuppression is essential to optimize graft preservation. As with our patient, this may be achieved with sirolimus, which has both immunosuppressive and anti-tumor effects, and has also been shown to reduce the risk of PTLD in liver (29) and heart (30) transplant recipients. Our patient demonstrates that this may aid in the prevention of both rejection and recurrence of PTLD in VCA patients.

Emerging PTLD interventions include chimeric antigen receptor-T cell (CAR-T) therapy, which has been shown to be effective for refractory PTLD (31). In addition, agents including ofatumumab, a CD20 monoclonal antibody, and brentuximab vedotin, a CD30-directed antibody-drug conjugate, have shown promise in case reports (32). Unlike SOT or HSCT recipients whose transplants are critical for survival, VCA patients may alternatively choose to pursue re-amputation. This should be considered when the reduction of immunosuppression precipitates rejection, or when PTLD poses an imminent threat since it can lead to complete remission, as in the PCNS patient (11). When weighing this treatment option, clinicians need to be cognizant of the unique ethical considerations of VCA explantation. While potentially lifesaving, graft removal is likely to cause emotional and psychological distress to patients who have undergone intensive preparation, lengthy surgical procedures, and taxing recoveries. In addition, re-amputation will likely feel alienating to patients, especially for those with late or very late onset PTLD who have successfully integrated their allotransplantations into their everyday lives, and concurrently their self-image, after a long period of incorporation. Given the low likelihood of ever receiving a successful second allograft, it will be difficult for patients to consent to graft removal when other options are available.

Though our patient did not pursue explantation, his aggressive PTLD subtype and high disease burden warranted a combined reduction of immunosuppression plus immunochemotherapy, resulting in complete remission while sparing the allograft. Our patient's thromboembolism required prolonged anticoagulation, highlighting unique layers of management complexity in VCA patients, including monitoring and treating cardiovascular complications.

The limitations of our treatment strategy include the omission of novel agents that may have increased efficacy in select patients; however, our multimodal approach facilitated complete remission without compromising the allograft.

With the increasing frequency of VCA, it is likely that we will continue to see more cases of VCA-related PTLD. It is important for clinicians to recognize this potentially life-threatening consequence of VCA so that they can thoroughly engage in risk-benefit discussions with patients. Perhaps, more importantly, recognizing this risk will help speed up the diagnosis and treatment in VCA patients. Understanding the potentially unique presentations in these patients and therapeutic options, such as altered immunosuppression and allograft removal, will lead to improved long-term outcomes.

## Data Availability

The original contributions presented in the study are included in the article/Supplementary Material, further inquiries can be directed to the corresponding author.
